# Mechanoepigenetics

**DOI:** 10.3389/fcell.2016.00113

**Published:** 2016-10-14

**Authors:** Yannis F. Missirlis

**Affiliations:** Department of Mechanical Engineering and Aeronautics, University of PatrasPatras, Greece

**Keywords:** mechanical signals, chromatin remodeling, mechanoepigenetics, mechanotransduction, stem cell fate

Life in our planet has originated, evolved, and adapted in specific chemical and physical environments, which also changed locally or globally due to the activities of all living systems.

Among the forces that influence biological functions, such as electromagnetic, thermal and mechanical ones, gravity, quantified by the universal constant **g**, is everpresent. It is not surprising, therefore, that mechanical forces influence many, if not all, biological processes. Changes in the value of **g** therefore might lead to dramatic physiological or epigenetic changes (Pardo et al., 2005; Singh et al., 2010).

Forces are not observable but their action, that is deformations, are. At the whole organism and tissue level the science of biomechanics produced very useful results in understanding the physiology and pathology of almost all the tissues, organs and the whole organism. In the last 50 years biomechanics extended its research subject to include cells and subcellular components using appropriate tools, such as micropipette aspiration, atomic force microscopy, optical tweezers, et.al, mainly *in-vitro*. As imaging and force manipulating techniques, which would reveal events with spatiotemporal resolution at the nano- and pico-meter scale, have come into use recently new knowledge on how cellular processes work is emerging in the literature (Seo et al., 2016). While all of these new findings are exciting they have been mainly obtained using *in-vitro* systems. Indeed the challenges for applying those techniques in living cells *in-vivo* are not trivial (Dufrene et al., 2011).

The force-deformation process results in what is known as mechanical signals. Mechanical signals are of different modalities, known as local strains due to tension, compression, bending, torsion, or shear rate due to flow, or combinations thereof. When one or more of such strains are sensed by the specialized cellular mechanosensors, such as integrins at the basal cell-extracellular matrix (ECM) interface, or strain sensitive ionic channels at the cell's apical surface, or junction proteins at cell-cell interface, mechanobiological events are initiated, which cross into the cytoplasm.

What are the exact events that take place during the transmission of the mechanical signals from, for example the integrin—ECM molecular junction, through the integrin(s), through the cellular membrane and into the inside end point of the integrin(s) is under intense investigation. Indeed, force is a signal that cells cannot ignore (Yusko and Asbury, 2014).

The “biochemical dogma” up until very recently was that a series of biochemical reactions start at the junction of integrins and a system of proteins in that neighborhood, like talin, vinculin, several kinases et al. A cascade of these reactions is thought to transmit the external (mechanical) information through the cytoplasm into the nucleus of the cell and, somehow, reach and interact with specific parts of the DNA.

Recent evidence however demonstrates that, concurrently with the biochemical cascade messages, mechanical signals from outside the cells travel all the way to the chromatin complex inside the nucleus, the process called mechanotransduction (Isermann and Lammerding, 2013; Swift and Discher, 2014). This direct mechanical route involves the cytoplasmic filamentous proteins actin, tubulin and intermediate fibers, connected with nesprins, which through nuclear envelope complexes transfer the mechanical signals to lamins. Lamins connect to the chromatin complex.

A plausible question maybe asked then: what is the effect of the external mechanical signal on the chromatin? A suggestion is that such signals are rapidly transmitted to the genome (Haase et al., 2016) and the chromatin structure is remodeled, is decondensed, specifically at transcription sites (Wang et al., 2014). The implication therefore is that by mechanically deconvoluting the chromatin complex there will be free passages for the appropriate biomolecules that have been recruited in the neighborhood to access the right sites at the DNA molecule.

Recapitulating the above, as a first order approximation, I suggest that mechanical signals originating outside a cell (as an imposed external force, or as a response of the cell interrogating the stiffness of its substrate or neighboring cell, or as an internally generated contraction force) travel on dynamic vehicles. These vehicles may be transmembrane complexes (integrins, laminin/dystrophins) connected to actin/tubulin/intermediate filaments, which in turn, through the LINCs transnuclear envelope system (Jahed et al., 2014) and the lamins, effect remodeling of the chromatin. Figure 1 depicts the above.

**Figure 1:**
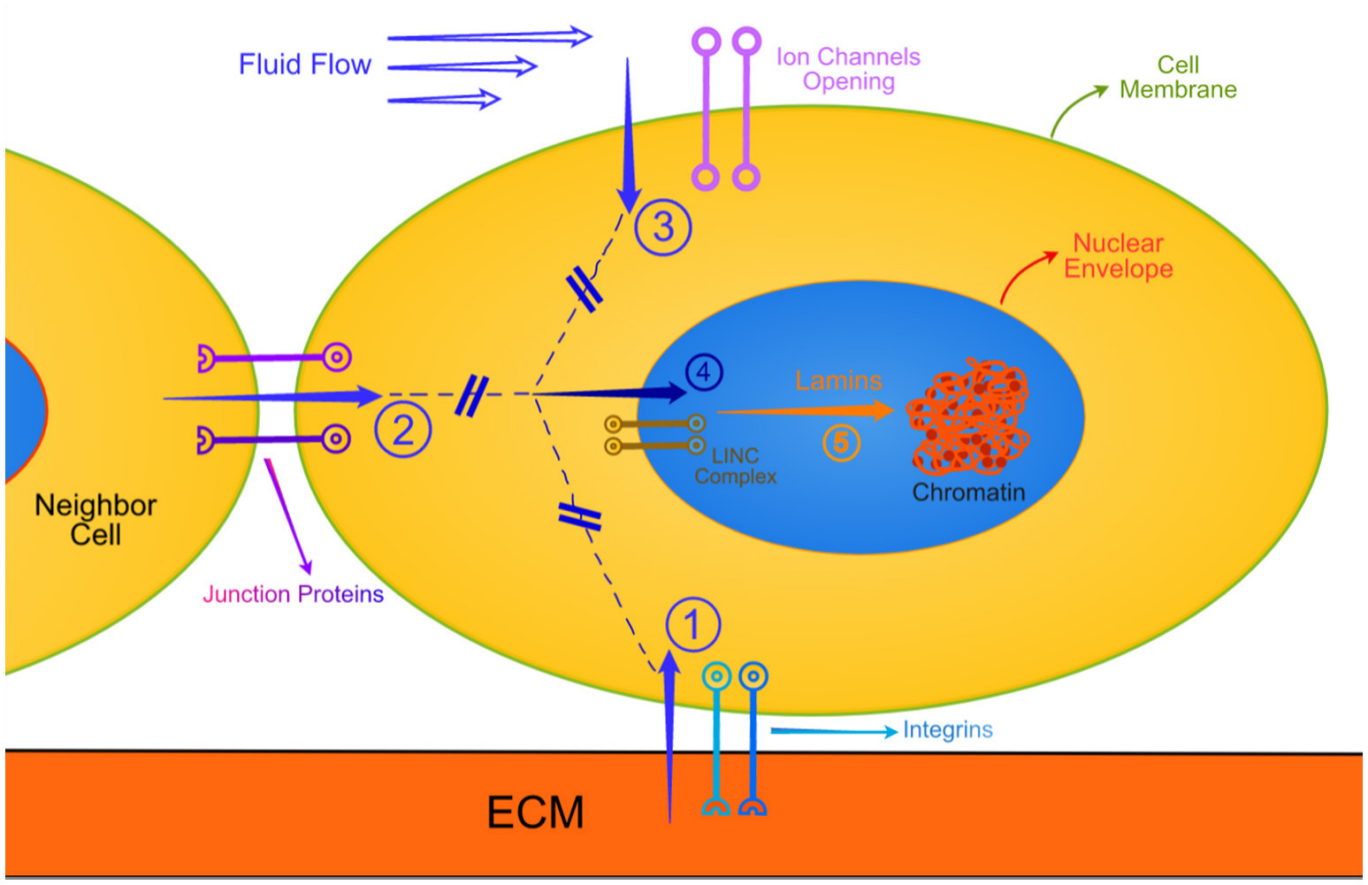
**This is a very simplified schematic of the various routes that external mechanical signals travel all the way to the chromatin**. Similar biochemical routes are not shown for simplicity. Also not shown are the interactive modulation of the mechanotransduction by biochemical entities. The cell is in contact with extracellular matrix (ECM), a neighbor cell, and exposed to a flowing biological fluid. Arrow 1: Typical focal adhesions subjected to tension, compression, torsion, bending or combination thereof. Arrow 2: Similar mechanical deformations of junction protein complexes. Arrow 3: Ion channels open to varying degree of shear rates due to pulsatile flows. The generated mechanical signals travel through the cytoplasm (broken lines) along mechanotransduction pathways, described in the text, and through the LINC complex (involving nesprins) transverse the nuclear envelope (Arrow 4). The messages emanating through arrow 4 engage mechanically the various lamins, which deform and, being connected to specific sites with chromatin, change the structural state of chromatin.

Now, this description of linearly sequential events, being a first approximation, is by default only scratching the surface of the complex processes going on. However, it serves the purpose of emphasizing that mechanical signals travel from outside the cell for at least one purpose. That is to remodel, to untangle the tightly packed chromatin, so that diffusion of relevant molecules is facilitated.

As the origin of mechanotransduction is to be found outside the cell, mainly at the adhesion sites of cells with the ECM or at cell-cell junctions, several remarks are of importance here. Firstly, the adhesions are dynamic structures, some having an inherent transient nature (Hanein and Horwitz, 2012) therefore they generate time-varying mechanical signals. Whether there is a specific threshold for a particular signal to generate mechanical information reaching the chromatin is unknown. Indeed there are many players involved in the cytoleskeletal force transmission complex, one publication citing at least 37 (Luo et al., 2013). Secondly, there are many different types of mechanical perturbations, varying in amplitude and frequency, which enter the cell. As the cells grow, migrate, differentiate, and “follow their fate” the interrogation of their focal adhesions, chemically, structurally and mechanically, continues from embryogenesis through development, in health and disease until apoptosis (Geiger et al., 2009; Janmey and Miller, 2011; Mammoto et al., 2013; Missirlis et al., 2016).

There are several checkpoints from the moment a mechanical signal transverses the cellular membrane. Some of them are related to actin and associated proteins. It seems that actin is both a mechanical signal sensor (Galkin et al., 2012), a regulator (along with cofilin) of transcriptional coactivators related to growth (Aragona et al., 2013) in addition to its being a primary cytoskeletal force transmitting filament. Concomitantly there are cooperative actions between biochemical and mechanical signals, which may regulate remodeling of the actin cytoskeleton (Stachowiak et al., 2014). What is unknown up to now, is how the integration of the multitude of mechanical signals, being checked at some point along their route, relayed from one cytoskeletal system to another, cross-talking with biochemical signals along the way is sensed at the end point, by histones or chromatin or both.

While there has been a long debate on the definition of epigenetics (Holliday, 2006) it is fair to state that epigenetics is the sum of all mechanisms necessary for the unfolding of the genetic program for development. As a matter of fact it is epigenetics and not genetics that discriminates between different types of cells in an organism. The epigenetic information is heritable through cell divisions, for longer periods if the modification involves the genomic DNA rather than the histones. In all cases, it is processes such as methylation, acetylation, phosphorylation or ubiquitinylation at specific sites that are involved.

The proposed, then, term mechanoepigenetics refers exactly to the connection between mechanical signaling and epigenetic action on chromatin. This does not preclude other environmental cues of influencing epigenetic modifications, such as heat shock, exposure to heavy metals, UV light or specific oxidation conditions (Saksouk et al., 2015). However, it seems that the mechanical signals are necessary if not sufficient for preparing chromatin to be modified. Modifications that involve chromatin unfolding (Collepardo-Guevara et al., 2015) or sliding sheets of chromatin (Wang et al., 2014) suggest the action of force. An important property to be considered here also is the fact that mechanical signals, by their nature, travel much faster that biochemical ones (Forgacs, 1995). Hence they prepare, by a still not clearly understood process, the exposition of DNA sites for the appropriate biochemical interactions.

The importance of mechanoepigenetics becomes evident in health and disease. Morphogenesis and development all need a cohort of mechanical signals, either to direct the right movement of morphogens in a concentration dependent manner, or possibly indirectly by epigenetic changes (Tabata and Takei, 2004; Mammoto et al., 2013). Homeostasis and especially mechanical homeostasis is under intense investigation at the cellular level (Weng et al., 2016). It may be that deregulated epigenetic mechanisms result, to a great extent in numerous diseases and cancers, as the compaction (a clearly mechanical effect) status of pericentromeres, and the expression potential thereof are affected (Saksouk et al., 2015). A more thorough description on cancer and other diseases is out of the scope of the present article.

Mechanoepigenetics may play a role not only in, for example methylation of DNA at cytosine bases for silencing specific genes, but also in the reverse process: demethylation, in order to reactivate silenced genes. This could lead to the use of appropriate mechanical signaling to embryonic stem cells for their specialized differentiation. It is interesting that stem cells interrogate and respond not only to externally imposed or self-generated mechanical signaling but also to the mechanical state of their substrate, such as the stiffness or stress relaxation of tunable hydrogels (Caiazzo et al., 2016; Chaudhuri et al., 2016).

A final note on mechanoepigenetics is presented here in lieu of a concluding remark. While the reductionist approach has provided us with useful information and understanding on the probable way of the workings of nature, and in particular of the cell, it has severe limitations as “by nature” it focuses mostly on single events. The proposed model of combination of the collaborative work of mechanical and biochemical signal transmission to the chromatin, resulting in epigenetic modifications, i.e., mechanoepigenetics, is an attempt to move upwards toward the systemic understanding of cellular processes.

## Author contributions

The author confirms being the sole contributor of this work and approved it for publication.

### Conflict of interest statement

The author declares that the research was conducted in the absence of any commercial or financial relationships that could be construed as a potential conflict of interest. The reviewer CC and handling Editor declared their shared affiliation, and the handling Editor states that the process nevertheless met the standards of a fair and objective review. The reviewer ST declared a shared affiliation, though no other collaboration, with the author to the handling Editor, who ensured that the process nevertheless met the standards of a fair and objective review.
